# Electronic structures and enhanced optical properties of blue phosphorene/transition metal dichalcogenides van der Waals heterostructures

**DOI:** 10.1038/srep31994

**Published:** 2016-08-24

**Authors:** Qiong Peng, Zhenyu Wang, Baisheng Sa, Bo Wu, Zhimei Sun

**Affiliations:** 1 Multiscale Computational Materials Facility, College of Materials Science and Engineering, Fuzhou University, and Key Laboratory of Eco-materials Advanced Technology (Fuzhou University), Fujian Province University, Fuzhou 350100, P. R. China; 2 School of Materials Science and Engineering, and Center for Integrated Computational Materials Engineering, International Research Institute for Multidisciplinary Science, Beihang University, Beijing 100191, P. R. China

## Abstract

As a fast emerging topic, van der Waals (vdW) heterostructures have been proposed to modify two-dimensional layered materials with desired properties, thus greatly extending the applications of these materials. In this work, the stacking characteristics, electronic structures, band edge alignments, charge density distributions and optical properties of blue phosphorene/transition metal dichalcogenides (BlueP/TMDs) vdW heterostructures were systematically studied based on vdW corrected density functional theory. Interestingly, the valence band maximum and conduction band minimum are located in different parts of BlueP/MoSe_2_, BlueP/WS_2_ and BlueP/WSe_2_ heterostructures. The MoSe_2_, WS_2_ or WSe_2_ layer can be used as the electron donor and the BlueP layer can be used as the electron acceptor. We further found that the optical properties under visible-light irradiation of BlueP/TMDs vdW heterostructures are significantly improved. In particular, the predicted upper limit energy conversion efficiencies of BlueP/MoS_2_ and BlueP/MoSe_2_ heterostructures reach as large as 1.16% and 0.98%, respectively, suggesting their potential applications in efficient thin-film solar cells and optoelectronic devices.

Since the experimental discovery of graphene^1^, the two-dimensional (2D) materials have taken global interests owing to their distinguished physical and chemical properties, adaptability, as well as multi-functionality. Generally, 2D materials show markedly different electronic, optical, and catalytic properties compared to their bulk counterparts due to the quantum confinement effect. In recent years, pure elements 2D materials, such as black phosphorene^2^,^3^,^4^, silicene^5^, germanene^6^,^7^ and stanene^8^, as well as binary systems of monolayer hexagonal boron nitride^9^,^10^ (hBN), single-layer semiconducting transition metal dichalcogenides^11^,^12^ (TMDs), MXenes^13^,^14^ and so on are widely studied. Nowadays, the combinations of various 2D materials together raise the possibility of the designing of van der Waals (vdW) heterostructures^15^. These heterostructures possess significantly improved electronic and optical properties compared to 2D materials themselves due to the mutual interaction between the layers^16^. Till now, a great deal of efforts has been made to obtain many vdW heterostructures as the key materials for next generation energy and environment related devices. For instance, the graphene-based heterostructure shows improved electronic properties compared to the individual graphene^17^,^18^. Among them, the black phosphorene/graphene heterostructure presents high capacity in sodium-ion batteries^19^.

It is worth noting that the TMDs-based vdW heterostructures have obtained increasing interest due to their extraordinary electronic and optical properties. The electronic conductivity and photochemical performances are greatly enhanced in graphene/MoS_2_ heterojunction^20^,^21^. The black phosphorene/MoS_2_ p-n diode as a photodetector shows a photodetection responsivity of 418 mA/W, which is nearly 100 times higher than the reported black phosphorus phototransistor^22^. For WX_2_/MoX_2_ (X = S, Se, Te) junction, spontaneous charge separation occurs when excitons diffuse to the junction, which is needed for photovoltaics^23^. The MoS_2_/hBN heterostructure could serve as a prototypical example for band structure engineering of 2D crystals with atomic layer precision^10^. The MoS_2_/AlN and MoS_2_/GaN vdW heterostructures exhibit high-efficiency photocatalytic activity for water splitting^24^,^25^.

To date, the puckered structure of black phosphorene can be converted to a more symmetric buckled structure by certain dislocation of constituent phosphorus atoms, termed as blue phosphorene (BlueP)^26^. And the single-layer BlueP is nearly as thermally stable as monolayer black phosphorene^26^,^27^,^28^. Due to the fact that both BlueP and TMDs monolayers share the same hexagonal crystal structure^26^,^29^, it is possible to construct appropriate BlueP/TMDs vdW heterostructures. It is worth noting that the lattice parameter of BlueP matches with many TMDs (for example, MoS_2_, MoSe_2_, WS_2_ and WSe_2_) perfectly. In this regard, investigating the electronic and optical properties of BlueP/TMDs vdW heterostructures is anticipated and of great interest and importance. In this work, the structural, electronic and optical properties of BlueP/TMDs (TMDs = MoS_2_, MoSe_2_, WS_2_ and WSe_2_) vdW heterostructures were systematically studied using first-principles calculations based on the density functional theory (DFT). The band-decomposed charge density and optical spectra were evaluated to understand the nature of the bonding mechanism, charge transfer as well as the visible-light absorption ability of the BlueP/TMDs vdW heterostructures.

## Results

The lattice constants of monolayer BlueP, MoS_2_, MoSe_2_, WS_2_ and WSe_2_ were fully optimized and the values are 3.268, 3.164, 3.295, 3.165, 3.295 Å, respectively, which are in consistent with the reported data listed in Supplementary Table S1^24^,^26^,^27^,^30^,^31^,^32^. The interlayer lattice mismatches between BlueP and MoS_2_, MoSe_2_, WS_2_ and WSe_2_ were evaluated to be +3.18%, −0.82%, +3.15% and −0.82%, respectively, which are all in an acceptable range and accessible in experimental synthesis. For each BlueP/TMDs heterostructure, six most possible stacking configurations with six special rotation angles between the adjacent layers were explored, as shown in Supplementary Fig. S1. The rotation angles of BlueP monolayer with respect to TMDs are 0°, 60°, 120°, 180°, 240° and 300°, respectively. All systems are geometrically optimized for getting stable atomic configuration. The difference of total energy between the six different stacking configurations compared with the most stable one, the interlayer distance between monolayer BlueP and TMDs, as well as the *M* − *X* (*M* = Mo, W and *X* = S, Se) and P-P bond lengths for the BlueP/TMDs heterostructures are listed in Supplementary Table S2. According to the total energy data for each configuration and Eq. (1), configuration (a) is the most stable structure for all the heterostructures. Interestingly, with the rotation of the BlueP monolayer with respect to TMDs, we can find in Supplementary Table S2 that the total energy and interlayer distance of the heterostructures increase and maximize when the rotation angle reaches 180° (configuration (d)) for all the four heterostructures.

In order to investigate the thermodynamic stability of the BlueP/TMDs heterostructures, the formation energies according to Eq. (2) were calculated to be −165.6, −210.5, −161.2 and −209.7 meV/unit-cell for BlueP/MoS_2_, BlueP/MoSe_2_, BlueP/WS_2_ and BlueP/WSe_2_ heterostructures, respectively. The negative formation energies indicate thermodynamic stability and the possibility to obtain the BlueP/TMDs heterostructures experimentally. On the other hand, the binding energies^33^ between BlueP and TMDs monolayers in BlueP/MoS_2_, BlueP/MoSe_2_, BlueP/WS_2_ and BlueP/WSe_2_ heterostructures are 16.11, 19.46, 15.69 and 19.38 meV/Å^2^, respectively, which are in consistent with the typical vdW binding energy of around 20 meV/Å^2^ obtained by the advanced DFT calculations^34^. Therefore, the above BlueP/TMDs heterostructures belong to vdW heterostructures. Considering the fact that each P atoms occupies ~5 Å^2^ space of the vdW interface, the vdW binding between the BlueP and TMDs layers are slightly stronger than that of bilayer black phosphorus from the Quantum Monte Carlo study^4^.

The calculated electronic band structures of BlueP and TMDs monolayers are presented in Supplementary Fig. S2. It is seen that single-layer BlueP is an indirect gap semiconductor with the conduction band minimum (CBM) located between the Γ (0, 0, 0) and Μ (0, 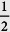, 0) points while valence band maximum (VBM) located between the Κ (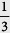, 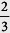, 0) and Γ points. The magnitude of the band gap of BlueP is 2.02 eV using optB86b-vdW, which agrees well with the literature values using GGA and DFT-D2 methods (see Supplementary Table S1)^26^,^27^. The band structures for TMDs monolayers are shown in Supplementary Fig. S2(c–f). It is seen that all TMDs monolayers exhibit direct gap characteristics because all of the CBM and VBM are located at the K point of the hexagonal first Brillouin zone. Furthermore, both VBM and CBM of TMDs mainly originate from the *d* orbital electrons of the transition metals. The calculated band gap values of monolayer MoS_2_, MoSe_2_, WS_2_ and WSe_2_ using optB86b-vdW are 1.77, 1.50, 1.89 and 1.63 eV, respectively, which agree well with previous work (see Supplementary Table S1)^32^,^35^,^36^,^37^,^38^.

**Figure 1:**
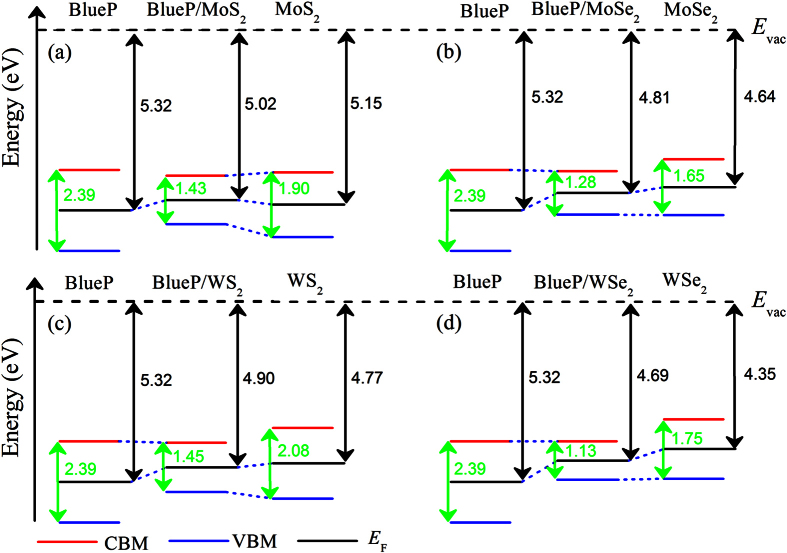
Band alignments and work functions of monolayers and heterostructures using hybrid functional, referring to the vacuum level (*E*_vac_). The black dashed lines illustrate *E*_vac_; the black double-headed arrows and numbers are the calculated work functions *W*, and the green double-headed arrows and numbers are hybrid functional predicted band gap.

To understand the band offset nature of BlueP/TMDs vdW heterostructures, the band edge alignments, the total density of states (DOS) and orbital-resolved partial DOS were studied systematically, as illustrated in Fig. 1 and Supplementary Fig. S3. For getting self-consistent results, we evaluated the accurate band gap values by the hybrid-DFT method with 8% Hartree-Fock exchange energy based on the optB86b-vdW approach for all the monolayers and heterostructures, which exactly reproduces the experimental gap of monolayer MoS_2_. The calculated band gap using hybrid functional calculations for BlueP, MoS_2_, MoSe_2_, WS_2_ and WSe_2_ monolayers are 2.39, 1.90, 1.65, 2.08 and 1.75 eV, respectively. These results are in good agreement with previous experimental data^11^,^30^,^38^ and theoretically reported values, as summarized in Supplementary Table S1^24^,^32^,^35^,^37^. As seen in Fig. 1, the band edge positions of these vdW heterostructures are located between CBM of BlueP and VBM of TMDs except for BlueP/MoS_2_ heterostructure. The band gaps of BlueP/MoS_2_, BlueP/MoSe_2_, BlueP/WS_2_ and BlueP/WSe_2_ heterostructures using hybrid functional are 1.43, 1.28, 1.45 and 1.13 eV, respectively. As summarized in Supplementary Table S3, the band gaps of heterostructures are smaller than the corresponding TMDs monolayers, indicating that the forming of vdW heterostructure reduces the band gap values. From Supplementary Fig. S3, it is clearly seen that the VBM is mainly occupied by the Mo-4*d* or W-5*d* electrons in these four vdW heterostructures. In contrast, the CBM primarily originates from the P-3*p* electrons for BlueP/MoSe_2_, BlueP/WS_2_ and BlueP/WSe_2_ heterostructures; while for BlueP/MoS_2_ heterostructure, the CBM is mainly contributed by 4*d* electrons of Mo atoms. It is consistent to the band edge alignments analysis. As a result, the Fermi level (*E*_F_) of BlueP/TMDs vdW heterostructures shifts and appears between the CBM of BlueP and VBM of TMDs. The work function of a material is a critical parameter commonly used as an intrinsic reference for band alignment^37^. It is seen from Fig. 1 that the predicted work functions of BlueP/MoS_2_ heterostructures are smaller than pristine BlueP and MoS_2_ monolayers; whereas, the work functions of BlueP/MoSe_2_, BlueP/WS_2_ and BlueP/WSe_2_ heterostructures are smaller significantly than pristine BlueP but larger slightly than TMDs. Owing to the energy of the Se-4*p* orbital higher than S-3*p* orbital, the work functions of BlueP/*M*Se_2_ (*M* = Mo, W) heterostructures are smaller than BlueP/*M*S_2_ heterostructures.

**Figure 2:**
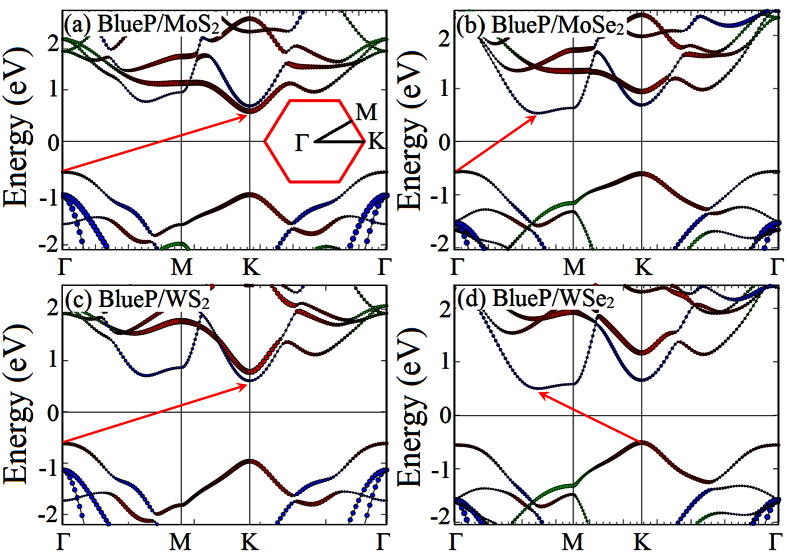
Band structures of (**a**) BlueP/MoS_2_, (**b**) BlueP/MoSe_2_, (**c**) BlueP/WS_2_ and (**d**) BlueP/WSe_2_ heterostructures using optB86b-vdW. The Fermi energy is set to 0 eV. The size of the red, green and blue circles illustrates the projected weight of *M*-*d* (*M* = Mo, W), *X*-*p* (*X* = S, Se) and P-*p* electrons, respectively.

From the calculated band structures in Fig. 2, it is seen that all the BlueP/TMDs vdW heterostructures are identified as indirect band gap semiconductors owing to the indirect band gap nature of BlueP and the interlayer coupling effect in the heterostructures. The BlueP/*M*S_2_ heterostructures can be devided into two groups. Group I includes the BlueP/MoS_2_ and BlueP/WS_2_ heterostructures (showing in Fig. 2(a,c)), where VBM is located at the Γ point and CBM is located at the K point. This is due to the fact that the BlueP monolayer shares similar valence band level at the Γ point and conduction band level at the K point with single layer MoS_2_ or WS_2_. The band interactions coming from the formations of the heterostructures pull up the energy level of the valence band at the Γ point and push down the energy level of the conduction band at the K point, which can also explain the variations of the band alignment as shown in Fig. 1(a,c). Group II involves the BlueP/MoSe_2_ and BlueP/WSe_2_ heterostructures (showing in Fig. 2(b,d)), where CBM is located between the Γ and Μ points. Although VBM is located at the Γ point for the BlueP/MoSe_2_ heterostructure, and which is located at the K point for the BlueP/WSe_2_ heterostructure, it is worth noting that the energy level of VBM at the Γ and K points are very close in these cases. For these heterostructures, the BlueP monolayer present deeper conduction and energy band levels than the MoSe_2_ or WSe_2_ layers. As a result, the interactions of the valence and conduction bands are weak between the BlueP monolayer and MoSe_2_ or WSe_2_ single layer. Hence the VBM keeps the main features of the single layer MoSe_2_ or WSe_2_, and CBM represents the features of the BlueP monolayer between the Γ and Μ points, which agrees well with the band alignment results in Fig. 1(b,d).

Considering the fact that there is a significant spin-orbit coupling (SOC) effect in TMDs^39^, we have calculated the band structures for all the five 2D monolayer materials and four heterostructures including the effect of SOC (illustrated in Supplementary Figs S4 and S5). It is seen that there is no significant SOC effect on band structure of BlueP. Otherwise, SOC splits off the double-degenerated transition metal (Mo or W) *d* electron occupied valence band state at the K point for the TMDs monolayers and BlueP/TMDs vdW heterostructures. As a results of the band splitting, the VBM shift to the K point for the BlueP/MoSe_2_ heterostructure. Apart from this, SOC slightly influences the predicted band structures without changing the characteristics and shapes of the band eigenvalues, which does not change the main results and conclusions of this work.

**Figure 3:**
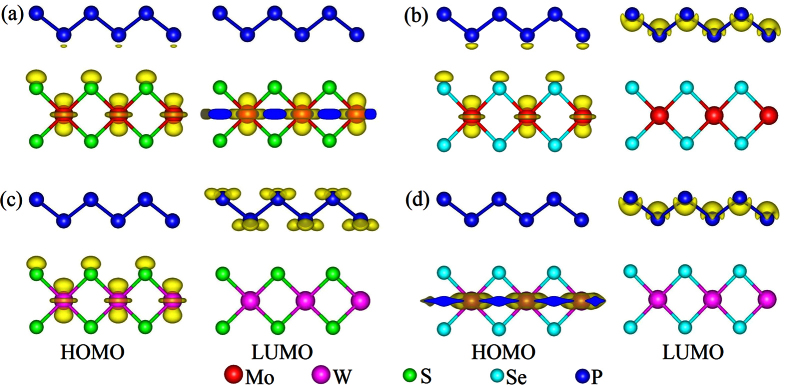
Band-decomposed charge density of the HOMO and LUMO for (**a**) BlueP/MoS_2_ and (**b**) BlueP/MoSe_2_, (**c**) BlueP/WS_2_ and (**d**) BlueP/WSe_2_ heterostructures with an isosurface value of 0.02 e/au^3^. The red, violet, green, cyan and blue balls are Mo, W, S, Se and P atoms, respectively.

**Figure 4:**
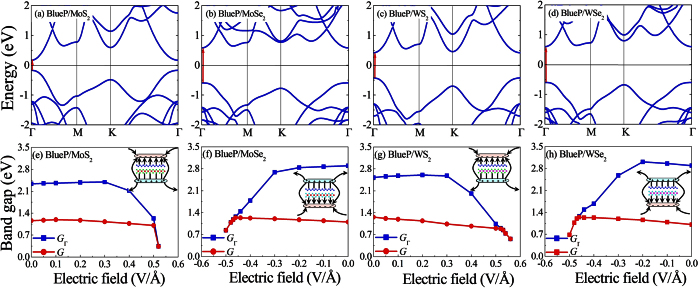
Indirect-direct band gap transition in BlueP/TMDs vdW heterostructures using optB86b-vdW. Electronic band structures of (**a**) BlueP/MoS_2_, (**b**) BlueP/MoSe_2_, (**c**) BlueP/WS_2_ and (**d**) BlueP/WSe_2_ heterostructures under external electric field of +0.52, −0.47, +0.53 and −0.47 V/Å, respectively; and (**e**–**h**) their corresponding band gap as a function of external electric fields. The Fermi energy is set to 0 eV. *G*_Γ_ is the calculated direct band gap at the Γ point and *G* is actual band gap under various external electric fields. The directions of the applied positive/negative electric field are shown in the insets.

The calculated band-decomposed charge density is shown in Fig. 3, which provides us a vividly picture to understand the role of the constituent layers in BlueP/TMDs vdW heterostructures. The decomposed charge density was calculated for the lowest unoccupied molecular orbital (LUMO) and the highest occupied molecular orbital (HOMO). The HOMOs are mainly consists of the Mo-4*d* or W-5*d* electrons, while the LUMOs are mainly contributed by the P-3*p* electrons in BlueP/MoSe_2_, BlueP/WS_2_ and BlueP/WSe_2_ heterostructures, except for BlueP/MoS_2_ heterostructure (its LUMO is mainly contributed by the Mo-4*d* electrons), which are in consistent with the analysis of the electronic structures. The results reveal that CBM and VBM are localized in different monolayers of BlueP/MoSe_2_, BlueP/WS_2_ and BlueP/WSe_2_ heterostructures, resulting in different spatial distribution of the lowest energy electron-hole pairs. Therefore, the MoSe_2_, WS_2_ or WSe_2_ layer can be potentially used as the electron donor and the BlueP layer as the electron acceptor in the corresponding heterostructures. The free electrons and holes can be effectively separated in the BlueP/MoSe_2_, BlueP/WS_2_ and BlueP/WSe_2_ heterostructures, indicating the potential applications of these vdW heterostructures for optoelectronics and solar energy conversion. Based on the distribution of charge densities, we found that the electrons are confined in the BlueP layer, while the holes in the TMDs side. Therefore, it is expected that spontaneous charge separation occurs when excitons diffuse to the BlueP/MoSe_2_, BlueP/WS_2_ and BlueP/WSe_2_ heterostructures, which is beneficial for the optoelectronic and photovoltaic applications^32^. We have calculated the frontier states to further study the interlayer interactions and hybridizations of the BlueP/*M*S_2_ heterostructures (see Fig. S6), which are beneficial for the microscopic understanding of the conduction channels^33^. We found that the HOMO/LOMO orbitals and frontier states show very similar characters. Comparing to the strong interlayer hybridizations in phosphorene/SiS heterostructures^40^, the interactions between the BlueP and *M*S_2_ layers are much weaker, which is expected in the vdW heterostructures. Very recently, Zhang *et al.* found that the indirect band gap nature in BlueP/MoS_2_ heterostructure can be tuned to direct by applying external electric fields^35^. We have confirmed the appearance of the indirect to direct band gap transition in the BlueP/MoSe_2_, BlueP/WS_2_ and BlueP/WSe_2_ heterostructures under −0.47, +0.53 and −0.47 V/Å external electric fields, respectively (see Fig. 4). Hence, the potential applications of these BlueP/TMDs vdW heterostructures are not restricted by their indirect band gap features.

The calculated plane-averaged electron density differences of BlueP/TMDs vdW heterostructures are shown in Supplementary Fig. S7 to better understand the nature of the bonding mechanism and the charge transfer between the BlueP and TMDs layers, where the magenta regions represent the charge accumulation, and the cyan regions represent depletion in the combined system relative to the two isolated components. As a result of the interlayer coupling effect, there is an obvious space charge accumulation region at the BlueP/TMDs heterointerfaces. In addition, the charge redistribution at the TMDs layer indicates that the formation of vdW heterostructures may affect the *M* − *X* bonds (*M* = Mo, W and *X* = S, Se).

**Figure 5:**
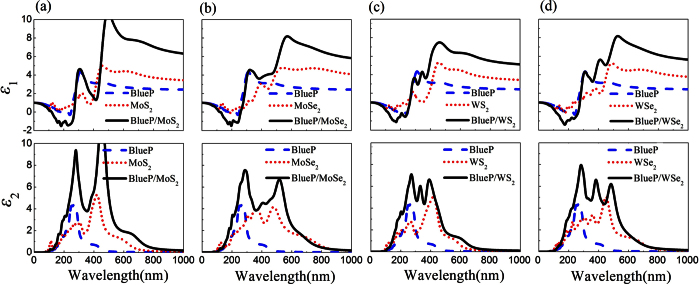
Real parts *ε*_1_ and imaginary parts *ε*_2_ of dielectric function versus wavelength via hybrid density functional calculations of (**a**) BlueP/MoS_2_, (**b**) BlueP/MoSe_2_, (**c**) BlueP/WS_2_ and (**d**) BlueP/WSe_2_ heterostructures (solid lines) compared to pristine BlueP (dashed lines) and TMDs monolayers (dotted lines).

To understand the effect of quantum confinement on the optical properties of BlueP/TMDs vdW heterostructures, the real (*ε*_1_) and imaginary (*ε*_2_) parts of the dielectric function were examined and compared to the dielectric function of corresponding single-layer BlueP and TMDs. Figure 5 presents the real parts *ε*_1_ and imaginary parts *ε*_2_ of dielectric function of the hybrid and single systems. For all the BlueP/TMDs vdW heterostructures, both the real and imaginary parts of the dielectric functions show larger values than the individual single systems (see Fig. 5). There are two distinguished peaks in UV light regions (200∼390 nm) and visible light regions (390∼770 nm) from the imaginary parts of dielectric functions, indicating enhanced optical properties of all the BlueP/TMDs vdW heterostructures.

## Discussion

**Figure 6:**
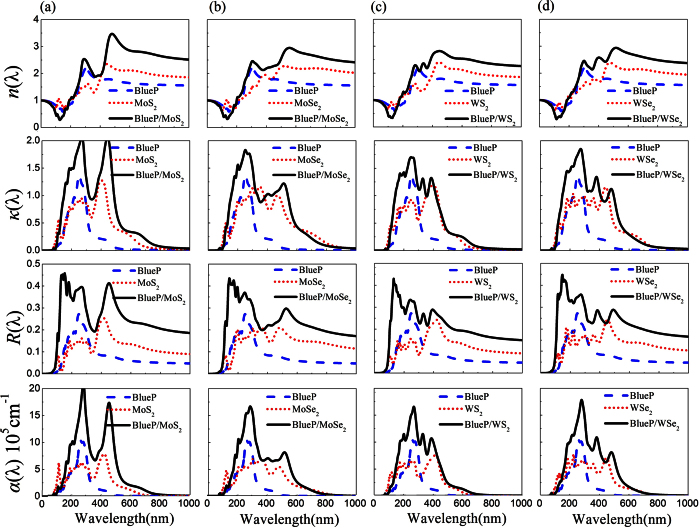
Optical properties of BlueP/TMDs vdW heterostructures. The optical refractive index *n*(*λ*), extinction coefficient *κ*(*λ*), reflectivity coefficient *R*(*λ*) and absorption coefficient *α*(*λ*) versus wavelength of (**a**) BlueP/MoS_2_, (**b**) BlueP/MoSe_2_, (**c**) BlueP/WS_2_ and (**d**) BlueP/WSe_2_ heterostructures (solid lines) compared to pristine BlueP (dashed lines) and TMDs monolayers (dotted lines).

Figure 6 shows the optical refractive index *n*(*λ*), extinction coefficient *κ*(*λ*), reflectivity coefficient *R*(*λ*) and absorption coefficient *α*(*λ*) of BlueP/TMDs vdW heterostructures from the dielectric functions according to Eqs (6, 7, 8, 9). Since all the vdW heterostructures show very similar tendency of the optical properties, we present the BlueP/MoSe_2_ heterostructure as an example in detail (see Fig. 6(b)). The refractive index *n*(*λ*) of BlueP/MoSe_2_ below 300 nm is very close to that of BlueP, but is much higher than that of MoSe_2_ above 500 nm. It is seen that extinction coefficient *κ*(*λ*) of BlueP/MoSe_2_ heterostructure is generally enhanced, especially in UV light ranges. Moreover, the reflectivity coefficient *R*(*λ*) of BlueP/MoSe_2_ heterostructure is increased twice compared with the individual monolayers. More importantly, BlueP/MoSe_2_ heterostructure shows substantial absorption in both visible light and UV light areas, while BlueP monolayer shows only UV light absorption and MoSe_2_ monolayer shows absorption mainly in the visible light region. In addition, its absorption region around 300 and 500 nm is clearly enhanced. Generally speaking, it clearly shows that BlueP/TMDs vdW heterostructures are able to harvest the visible light and UV light more efficiently than the individual single systems. The similar phenomenon can be found in the hybrid GeH/graphene nanocomposite^41^. The heterostructure formation can not only improve the charge separation of photo-induced electron-hole pairs but also broaden the light absorption range^42^. Therefore, the BlueP/TMDs vdW heterostructures possess potential applications in efficient solar cells and ultrathin optoelectronic devices.

To go a step further, we introduced the estimation method by Shi *et al.*^43^,^44^ according to Eqs (10, 11, 12, 13) to calculate the theoretical upper limit conversion efficiency of sunlight for BlueP, TMDs monolayers and BlueP/TMDs vdW heterostructures. The corresponding results are listed in Table 1. It is reported that WSe_2_ monolayer has a strong optical absorbance and can be used to fabricate solar cells with light-to-electricity conversion efficiencies of ~0.5%^12^. As seen in Table 1 that our estimated efficiency for WSe_2_ with 0.32% agrees well with the experimental result, which verifies the reliability and authenticity of this work. Theoretical calculations also predict that the BlueP/TMDs vdW heterostructures have consistently larger conversion efficiencies than the corresponding individual single systems due to the larger overlap between their absorbance and the solar spectrum. In particular, the predicted upper limit energy conversion efficiencies of BlueP/MoS_2_ and BlueP/MoSe_2_ heterostructures reach as large as 1.16% and 0.98%, respectively, which are comparable to the graphene/MoS_2_ ultrathin heterostructure solar cell with ~1% energy conversion efficiency^45^. The semiconductor solar cell, as a green and efficient technology, has gained wide attention because of its promising applications in solar energy utilization and pollutant elimination. The proposed BlueP/TMDs vdW heterostructures not only exhibit higher energy conversion performance than the individual 2D TMDs monolayers, but also present many distinguished properties, such as good stability, moderate band gap, indirect-direct band gap transition, electronic-hole separation as well as fascinating light adsorption. Therefore, these vdW heterostructures break the limitation of the individual 2D materials, which provide more choices for the scientist and engineer to design the next generation solar cell devices.

## Conclusion

In summary, we have systematically studied the stacking configurations induced electronic structures, band edge alignments, charge density distributions and optical properties of BlueP/TMDs vdW heterostructures based on vdW corrected density functional theory. The negative formation energies as well as the good lattice match protect the thermodynamic stability of the BlueP/TMDs vdW heterostructures. All the BlueP/TMDs vdW heterostructures exhibit indirect gap characteristics. The VBM and CBM are localized in different parts of the BlueP/MoSe_2_, BlueP/WS_2_ and BlueP/WSe_2_ heterostructures. The MoSe_2_, WS_2_ or WSe_2_ layer can be used as the electron donor and the BlueP layer can be used as the electron acceptor in the corresponding heterostructures. The BlueP/TMDs vdW heterostructures exhibit unusually stronger optical absorbance and energy conversion efficiency in the visible range. The results in this work provide a fundamental understanding and guideline for designing high performance optoelectronic and photovoltaic devices.

## Methods

### Density functional theory calculations

Our calculations were performed based on the density functional theory (DFT) in conjunction with the projector-augmented-wave (PAW) potential as implemented in the Vienna ab initio Simulation Package (VASP)^46^,^47^. For the exchange-correlation energy, the Perdue-Burke-Ernzerhof (PBE) version of the generalized gradient approximation (GGA) was used^48^. The van der Waals density functional (vdW-DF) of optB86b were considered for all the calculations^49^,^50^. The valence electron configurations for P, S, Se, Mo and W were 3*s*^2^3*p*^3^, 3*s*^2^3*p*^4^, 4*s*^2^4*p*^4^, 4*d*^5^5*s*^1^ and 5*d*^4^6*s*^2^, respectively. A plane wave cutoff energy of 600 eV was used for the plane-wave expansion of the wave function. The first Brillouin zone was sampled with a fine grid of 7 × 7 × 1 for structure optimization and 15 × 15 × 1 for static calculation^51^. A vacuum of 30 Å along the *z* direction (perpendicular to the BlueP/TMDs vdW heterostructures layers) was constructed to eliminate the interaction with spurious replica images. The atomic positions were fully relaxed until satisfying an energy convergence of 10^−5^ eV and force convergence of 0.01 eV/Å. Due to the fact that the PBE functional underestimates the band gaps of semiconductors, the accurate band gap values and optical properties were further calculated by the hybrid functional approach. As seen in Table S1 and Fig. S8, the standard optB86b-vdW functional underestimates the band gap of well-known monolayer MoS_2_ by ~0.13 eV. Comparing to the standard HSE06 hybrid functional with 25% Hartree-Fock exchange energy^52^, we found that the 8% short range Hartree-Fock exchange energy based on optB86b-vdW can reproduce the experimental band gap of monolayer MoS_2_ and other TMDs without much deviation of the lattice parameter. Considering the fact that there is no experimental results available for the band gap of monolayer BlueP, we choose monolayer MoS_2_ as the reference to predict the band gap of the BlueP/TMDs vdW heterostructures. It is worth noting that the proportion of the short range Hartree-Fock exchange energy influences the absolute values of the predicted band gap only, without changing the variation tendency of the formation of the vdW heterostructures. To further check our results, we have calculated the quasiparticle band gaps of the monolayers and heterostructures using the G_0_W_0_ approximation^53^, which are listed in Tables S1 and S3 as well. As seen, our results agrees well with previously scGW_0_ results^54^. It is interesting to find in Fig. S8, the G_0_W_0_ approximation shows very similar band gap variation pattern to the hybrid functional calculations.

To determine the configuration of the heterostructure stacking, the energy difference Δ*E* was written as the following equation:


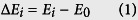


where *E*_0_ is the total energy of the most stable configuration, and *E*_*i*_ is the total energy of each configuration. To exam the stability of the heterostructures, the formation energy was obtained according to the following equation:^24^


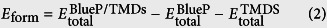


where 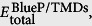 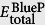 and 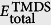 represent the total energies of BlueP/TMDs, BlueP and TMDs in one unit-cell, respectively. In addition, we have added the calculated equation of binding energy to evaluate the strength of the vdW force in BlueP/TMDs vdW heterostructures according to the following equation:


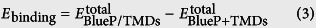


where 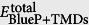 is the sum of the total energy of mutually independent single-layered BlueP and TMDs fixed in the corresponding heterostructure lattice. The work function *W* was obtained by the following expression:


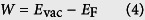


where *E*_vac_ is the energy of a stationary electron in the vacuum nearby the surface, *E*_F_ is the Fermi energy level. In addition, the plane-averaged electron density difference along the perpendicular direction to the interface was calculated according to the following equation:


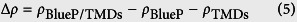


where the *ρ*_BlueP/TMDs_, *ρ*_BlueP_ and *ρ*_TMDs_ are the charge density of BlueP/TMDs vdW heterostructures, BlueP and TMDs systems, respectively.

### Optical properties

Optical properties are described by the photon wavelength dependent dielectric function *ε*(*λ*) = *ε*_1_(*λ*) + *iε*_2_(*λ*), which is mainly contributed from the electronic structures^55^. The refractive index *n*(λ) and the extinction coefficient *κ*(λ) are determined from *ε*_1_ and *ε*_2_ using^43^


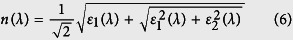


and


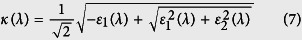


where *λ* is the photon wavelength. The reflectivity and absorption coefficient were calculated by^43^


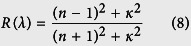


and


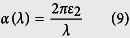


The estimated upper limit to the converted power *P* is based on the overlap between the solar spectrum and the absorbance:^44^


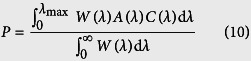


where *λ*_max_ is the longest wavelength that can be absorbed by ultrathin materials and is determined by the lowest-exciton energy *E*_g_ (i.e., the minimum band gap of the material),


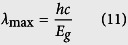


and *W*(*λ*) is the solar spectral irradiance at Air Mass 1.5 56. *A*(*λ*) is the directionally averaged absorbance of BlueP, TMDs monolayers and BlueP/TMDs vdW heterostructures according to^44^


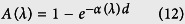


where *α* is the absorption coefficient, and *d* is the thickness of the simulation cell perpendicular to the layers. The term *C*(*λ*) is the conversion factor to account for the fraction of the photon energy converted to lowest-exciton energy (i.e., the thermalization loss).


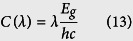


## Additional Information

**How to cite this article**: Peng, Q. *et al.* Electronic structures and enhanced optical properties of blue phosphorene/transition metal dichalcogenides van der Waals heterostructures. *Sci. Rep.* **6**, 31994; doi: 10.1038/srep31994 (2016).

## Supplementary Material

Supplementary Information

## Figures and Tables

**Table 1:** Upper limit of the energy conversion efficiency *P* (%) of sunlight to lowest-energy excitons for BlueP, TMDs monolayers and BlueP/TMDs vdW heterostructures.

System	BlueP	MoS_2_	MoSe_2_	WS_2_	WSe_2_	BlueP/MoS_2_	BlueP/MoSe_2_	BlueP/WS_2_	BlueP/WSe_2_
*P*_1_	0.02	0.27	0.37	0.25	0.32	1.16	0.98	0.62	0.72
Reference^12^					0.50				
