# Supplementary material for: Electronic structures and enhanced optical properties of blue phosphorene/transition metal dichalcogenides van der Waals heterostructures
Source: Sci Rep. 2016 Aug 24;6:31994. doi: 10.1038/srep31994 (PMC4995501; doi:10.1038/srep31994)
Supplement: Supplementary Information [file srep31994-s1.doc]

Supporting Information

**Electronic structures and enhanced optical properties of blue phosphorene/transition metal dichalcogenides van der Waals heterostructures**

Qiong Peng^1^, Zhenyu Wang^1^, Baisheng Sa^1,*^, Bo Wu^1,**^, Zhimei Sun^2,***^

^1^*Multiscale Computational Materials Facility,* *College of Materials Science and Engineering, Fuzhou University,* *and Key Laboratory of Eco-materials Advanced Technology (Fuzhou University), Fujian Province University, Fuzhou 350100, P. R. China*

^2^*School of Materials Science and Engineering, and Center for Integrated Computational Materials Engineering, International Research Institute for Multidisciplinary Science,* *Beihang University,* *Beijing 100191,* *P. R. China*

*Corresponding author: bssa@fzu.edu.cn.

**Corresponding author: wubo@fzu.edu.cn.

***Corresponding author: zmsun@buaa.edu.cn.

**Table S1.** **The lattice constants *a* (Å), sheet thickness *h* (Å), the *M-X* (*M* = Mo, W and *X* = S, Se) and P-P bond length *L* (Å) and band gaps *G* (eV) with different methods.**

| System | Functional | *a*(Å) | *h*(Å) | *L*(Å) | *G*_direct_(eV) | *G*_indirect_(eV) | Reference |
| --- | --- | --- | --- | --- | --- | --- | --- |
| BlueP | GGA |  | 1.24 | 2.27 |  | 1.94 | [1] |
|  | DFT-D2 | 3.326 |  | 2.27 |  | 2 | [2] |
|  | OptB86b | 3.268 | 1.245 | 2.261 |  | 2.02 | This work |
|  | HSE |  |  |  |  | 2.73 | [1] |
|  | HSE |  |  |  |  | 2.39 | This work |
|  | G_0_W_0_ |  |  |  |  | 3.56 | This work |
| MoS_2_ | LDA |  |  |  | 1.76 |  | [3] |
|  | DFT-D2 | 3.19 |  | 2.41 | 1.64 |  | [4] |
|  | LDA | 3.13 | 3.12 | 2.39 | 1.86 |  | [5] |
|  | GGA | 3.19 | 3.13 | 2.42 | 1.67 |  | [5] |
|  | OptB86b | 3.164 | 3.143 | 2.409 | 1.77 |  | This work |
|  | HSE |  |  |  | 1.92 |  | [4] |
|  | HSE |  |  |  | 1.90 |  | This work |
|  | G_0_W_0_ |  |  |  | 2.92 |  | This work |
|  | scGW_0_ |  |  |  | 2.80 |  | [6] |
|  | Exp. |  |  |  | 1.9 |  | [7] |
|  | Exp. |  |  |  | 1.87 |  | [8] |
| MoSe_2_ | LDA | 3.25 | 3.32 | 2.51 | 1.63 |  | [5] |
|  | GGA | 3.33 | 3.35 | 2.55 | 1.44 |  | [5] |
|  | OptB86b | 3.295 | 3.352 | 2.536 | 1.50 |  | This work |
|  | HSE |  |  |  | 1.65 |  | This work |
|  | G_0_W_0_ |  |  |  | 2.47 |  | This work |
| WS_2_ | GW |  |  |  | 1.77 |  | [5] |
|  | GGA | 3.19 | 3.14 | 2.42 | 1.81 |  | [5] |
|  | OptB86b | 3.165 | 3.154 | 2.414 | 1.89 |  | This work |
|  | HSE |  |  |  | 2.08 |  | This work |
|  | G_0_W_0_ |  |  |  | 3.17 |  | This work |
|  | scGW_0_ |  |  |  | 3.11 |  | [6] |
| WSe_2_ | DFT-D2 |  |  |  | 1.19 |  | [3] |
|  | GGA | 3.32 | 3.36 | 2.55 | 1.55 |  | [5] |
|  | OptB86b | 3.295 | 3.369 | 2.541 | 1.63 |  | This work |
|  | HSE |  |  |  | 1.75 |  | This work |
|  | G_0_W_0_ |  |  |  | 2.65 |  | This work |
|  | Exp. |  |  |  | 1.64 |  | [9] |

**Table S2.** **Energy difference Δ*E_i_* (meV) between various configurations, lattice constants *a* (Å), the lowest energy configuration, the interlayer distance *d* (Å), as well as the *M-X* (*M* = Mo, W and *X* = S, Se) and P-P bond length in BlueP/TMDs vdW heterostructures optimized by optB86b-vdW.**

| System | Configuration | Δ*E_i_*(meV) | *a*(Å) | *d*(Å) | *L*_M−X_(Å) | *L*_P−P_(Å) |
| --- | --- | --- | --- | --- | --- | --- |
| BlueP/MoS_2_ | a | 0 | 3.206 | 3.608 | 2.418 | 2.238 |
|  | b | 1.832 | 3.207 | 3.597 | 2.420 | 2.239 |
|  | c | 20.998 | 3.208 | 3.711 | 2.419 | 2.238 |
|  | d | 70.639 | 3.204 | 3.748 | 2.418 | 2.237 |
|  | e | 67.474 | 3.204 | 3.692 | 2.418 | 2.238 |
|  | f | 3.186 | 3.209 | 3.605 | 2.419 | 2.239 |
| BlueP/MoSe_2_ | a | 0 | 3.290 | 3.683 | 2.534 | 2.269 |
|  | b | 7.976 | 3.292 | 3.687 | 2.536 | 2.270 |
|  | c | 26.143 | 3.292 | 3.845 | 2.535 | 2.269 |
|  | d | 77.836 | 3.287 | 3.830 | 2.534 | 2.268 |
|  | e | 74.332 | 3.287 | 3.805 | 2.534 | 2.267 |
|  | f | 0.818 | 3.293 | 3.625 | 2.535 | 2.269 |
| BlueP/WS_2_ | a | 0 | 3.205 | 3.633 | 2.422 | 2.238 |
|  | b | 3.819 | 3.206 | 3.659 | 2.423 | 2.238 |
|  | c | 22.120 | 3.206 | 3.793 | 2.422 | 2.237 |
|  | d | 67.717 | 3.203 | 3.729 | 2.422 | 2.238 |
|  | e | 64.416 | 3.203 | 3.707 | 2.422 | 2.237 |
|  | f | 5.011 | 3.206 | 3.645 | 2.422 | 2.237 |
| BlueP/WSe_2_ | a | 0 | 3.289 | 3.686 | 2.540 | 2.269 |
|  | b | 10.843 | 3.290 | 3.776 | 2.541 | 2.269 |
|  | c | 27.834 | 3.292 | 3.859 | 2.541 | 2.269 |
|  | d | 76.416 | 3.287 | 3.836 | 2.540 | 2.268 |
|  | e | 72.999 | 3.287 | 3.807 | 2.540 | 2.268 |
|  | f | 2.553 | 3.293 | 3.645 | 2.541 | 2.269 |

**Table S3.** **The calculated band gaps *G* (eV) for BlueP/TMDs vdW heterostructures.**

| System | *G*-PBE | *G*-SOC | *G*-HSE | *G*-G_0_W_0_ | Reference |
| --- | --- | --- | --- | --- | --- |
| BlueP/MoS_2_ | 1.16 | 1.17 | 1.43 | 1.98 | This work |
|  | 1.19 |  | 1.39 |  | [10] |
| BlueP/MoSe_2_ | 1.10 | 1.08 | 1.28 | 1.76 | This work |
| BlueP/WS_2_ | 1.26 | 1.29 | 1.45 | 2.03 | This work |
| BlueP/WSe_2_ | 1.01 | 0.81 | 1.13 | 1.82 | This work |


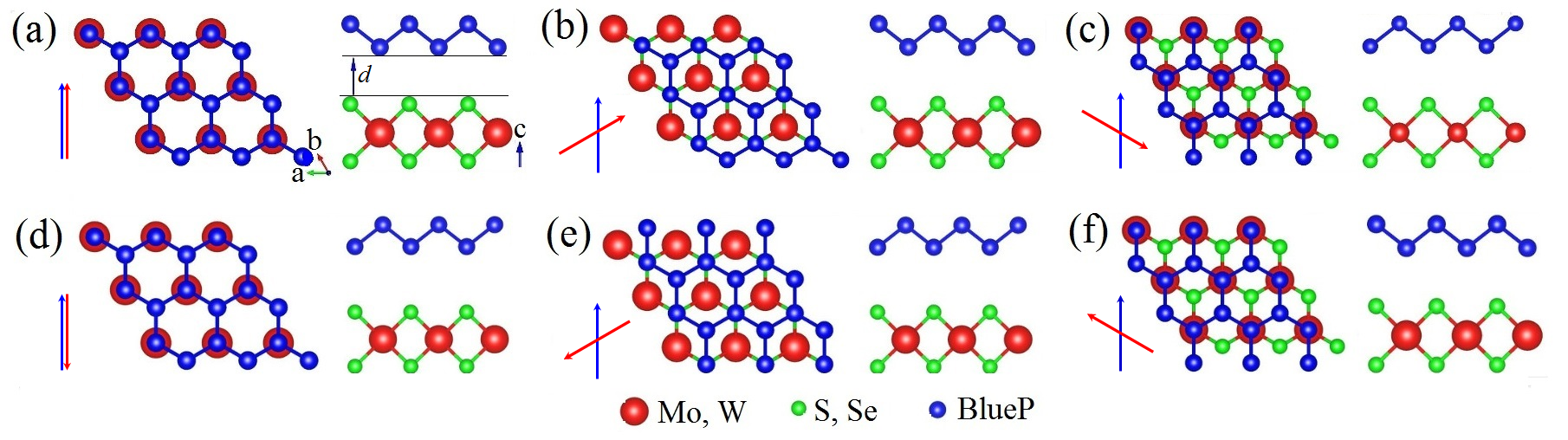


**Figure S1.** **Schematic views of the BlueP/TMDs vdW heterostructures with various stackings.** The rotation angles of BlueP monolayer with respect to TMDs are set to (**a**) 0°, (**b**) 60°, (**c**) 120°, (**d**) 180°, (**e**) 240°, and (**f**) 300°. Herein, the red, green and blue balls indicate the Mo (W), S (Se) and BlueP atoms, respectively.


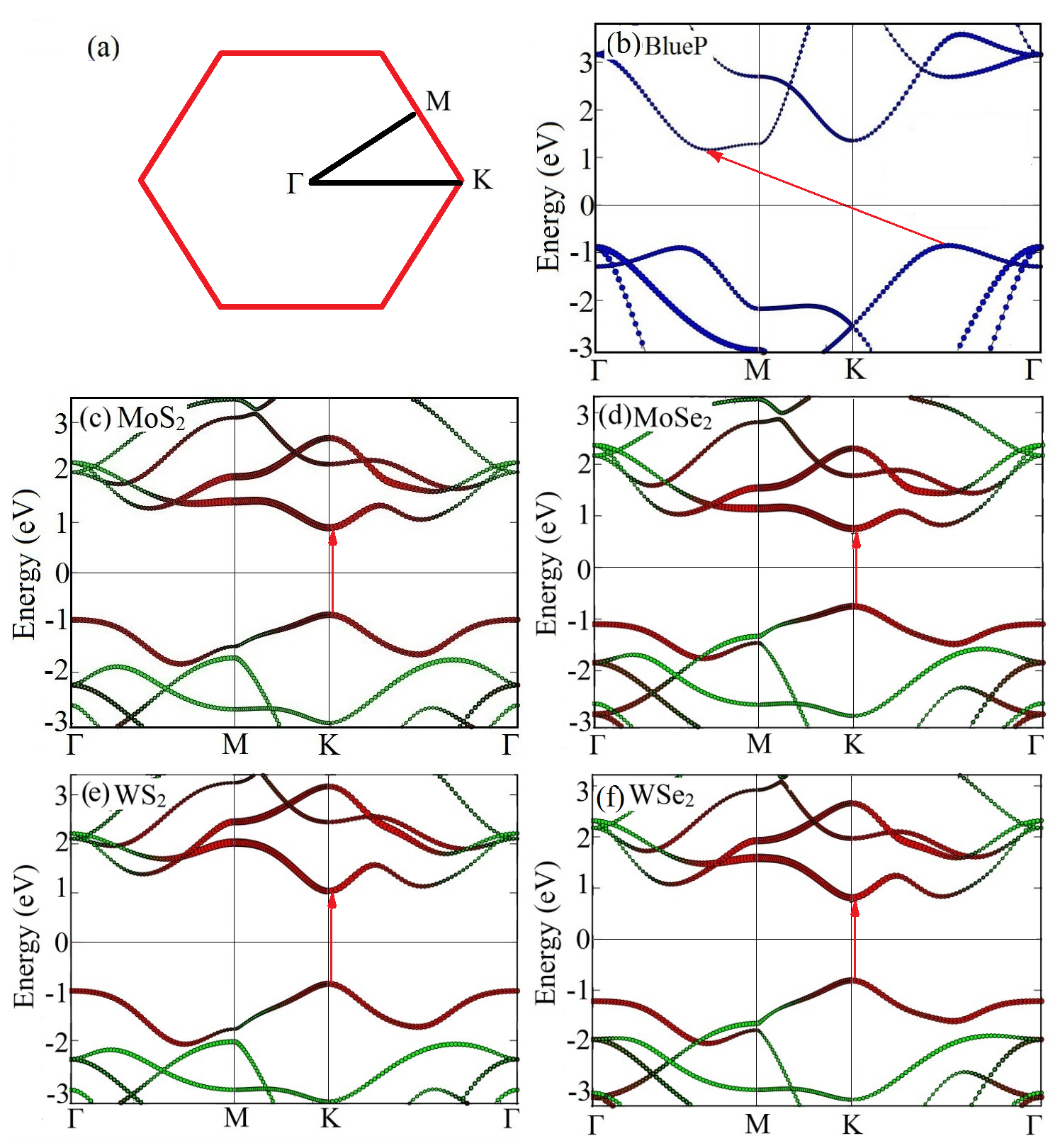


**Figure S2.** **Band structures of monolayers using optB86b-vdW.** (**a**) Brillouin zone with high-symmetry points labeled. Band structures of (**b**) BlueP, (**c**) MoS_2_, (**d**) MoSe_2_, (**e**) WS_2_ and (**f**) WSe_2_ monolayers. The Fermi energy is set to 0 eV. The size of the red, green and blue circles illustrates the projected weight of *M-d* (*M* = Mo, W), *X*-*p* (*X* = S, Se) and P-*p* electrons, respectively.


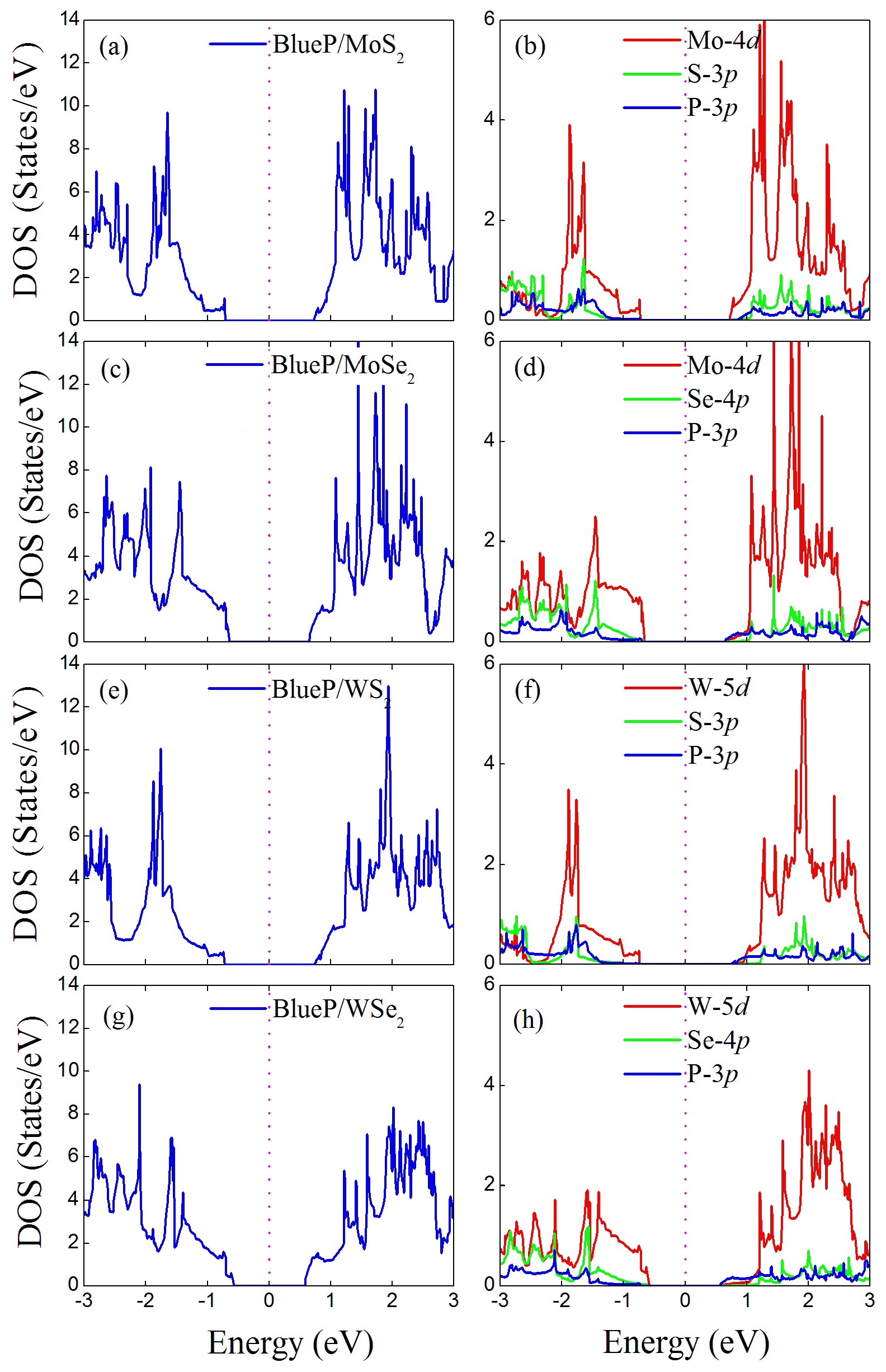


**Figure S3.** **The total density of states (DOS) and orbital-resolved partial DOS of (a,b) BlueP/MoS_2_, (c,d) BlueP/MoSe_2_, (e,f) BlueP/WS_2_ and (g,h) BlueP/WSe_2_ heterostructures using hybrid functional.** The vertical magenta dotted lines represent the Fermi level energy.


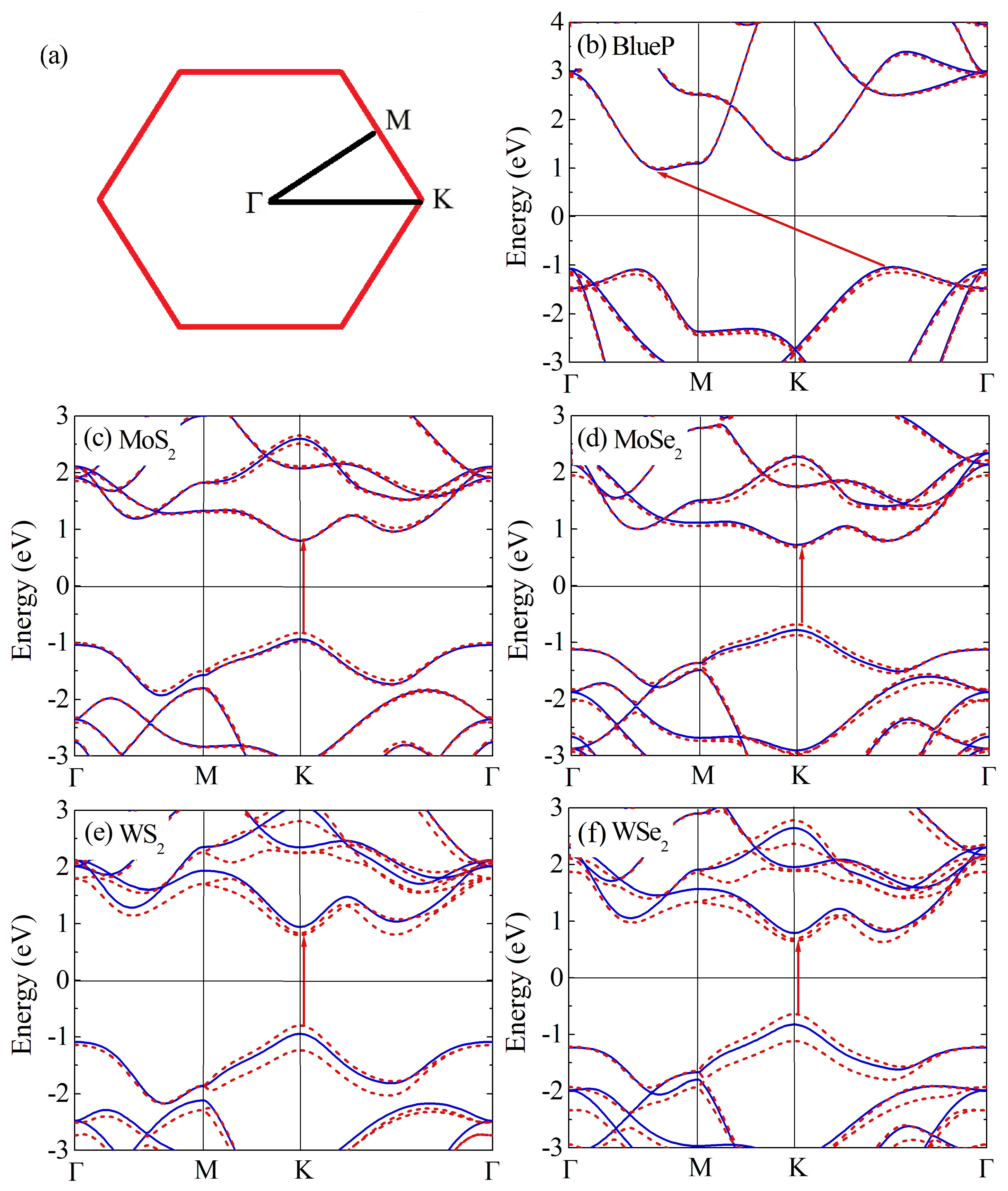


**Figure S4.** **Band structures of monolayers with (dashed line) and without (solid line) the spin-orbit coupling using optB86b-vdW.** (**a**) Brillouin zone with high-symmetry points labeled. Band structures of (**b**) BlueP, (**c**) MoS_2_, (**d**) MoSe_2_, (**e**) WS_2_ and (**f**) WSe_2_ monolayers. The Fermi energy is set to 0 eV.


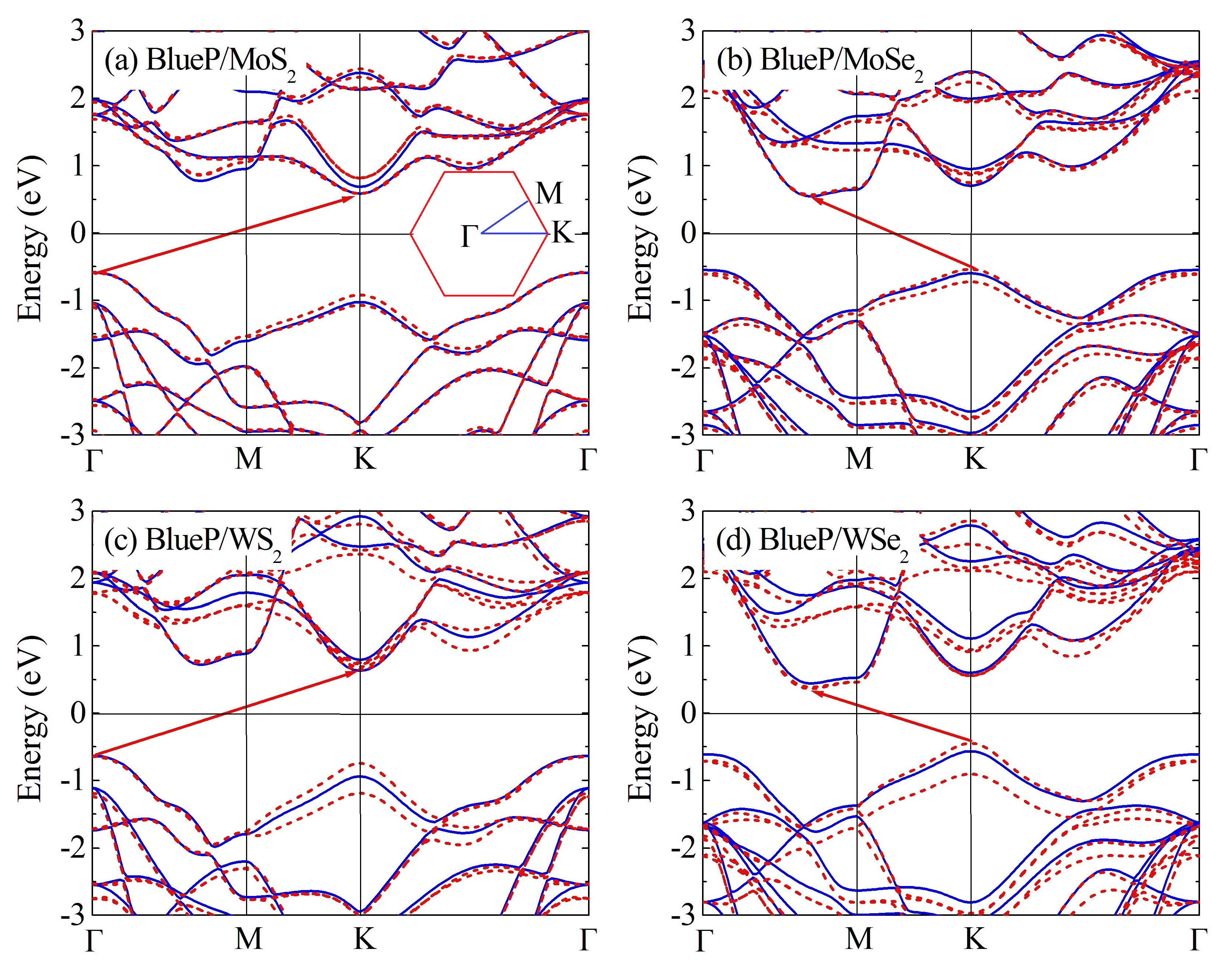


**Figure S5.** **Band structures of** **(a) BlueP/MoS_2_, (b) BlueP/MoSe_2_, (c) BlueP/WS_2_ and (d) BlueP/WSe_2_ heterostructures with (dashed line) and without (solid line) the spin-orbit coupling using optB86b-vdW, respectively.** The Fermi energy is set to 0 eV.


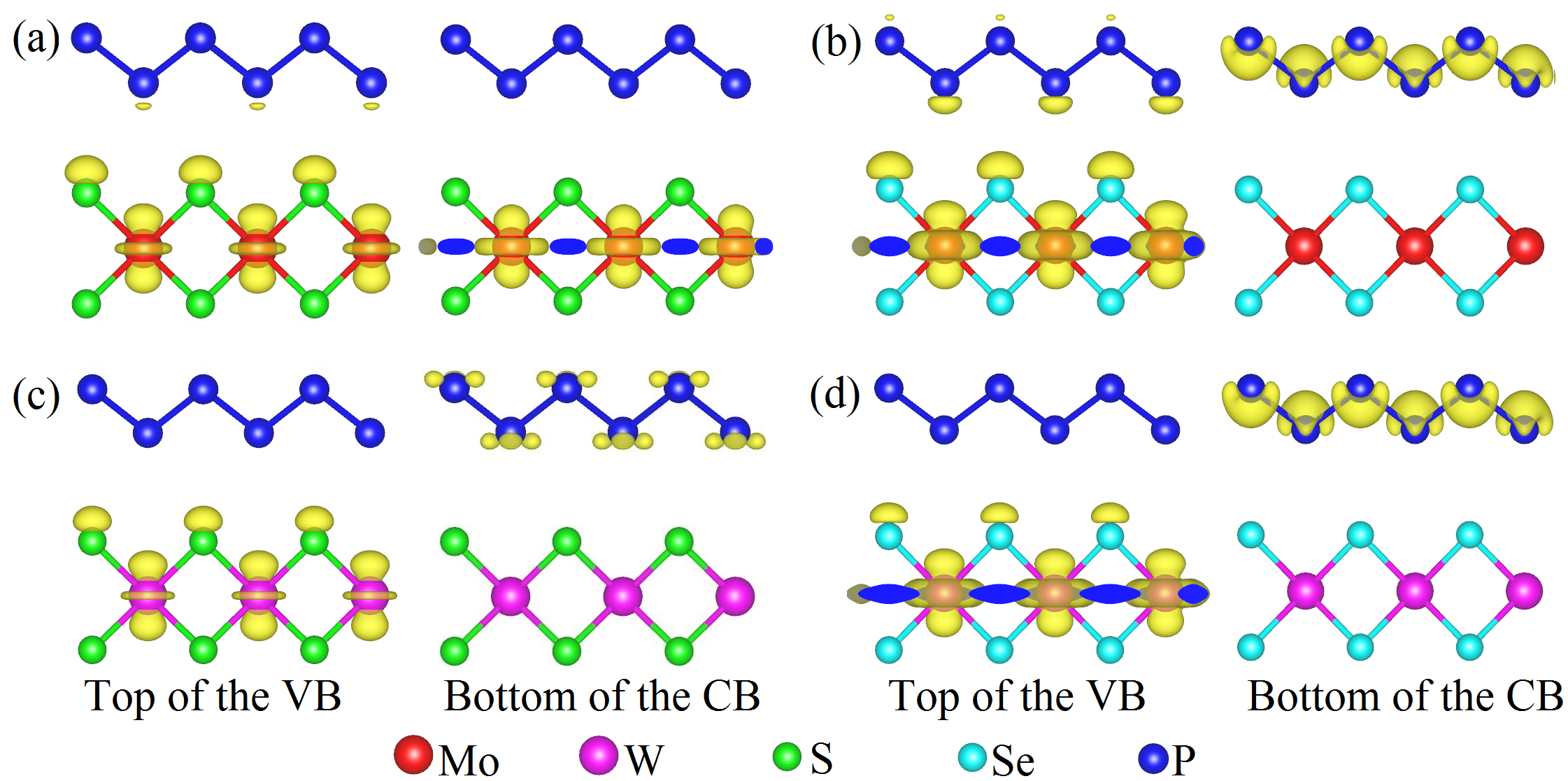


**Figure S6. Frontier states in the valence and conduction band range of (a) BlueP/MoS_2_ and (b) BlueP/MoSe_2_, (c) BlueP/WS_2_ and (d) BlueP/WSe_2_ heterostructures**. The energy range represented extends from the midgap to 0.1 eV below the top of the valence band (VB) for the valence frontier states and from the mid-gap to 0.1 eV above the bottom of the conduction band (CB) for the conduction frontier states. The isosurface values are 0.0025 e/au^3^.


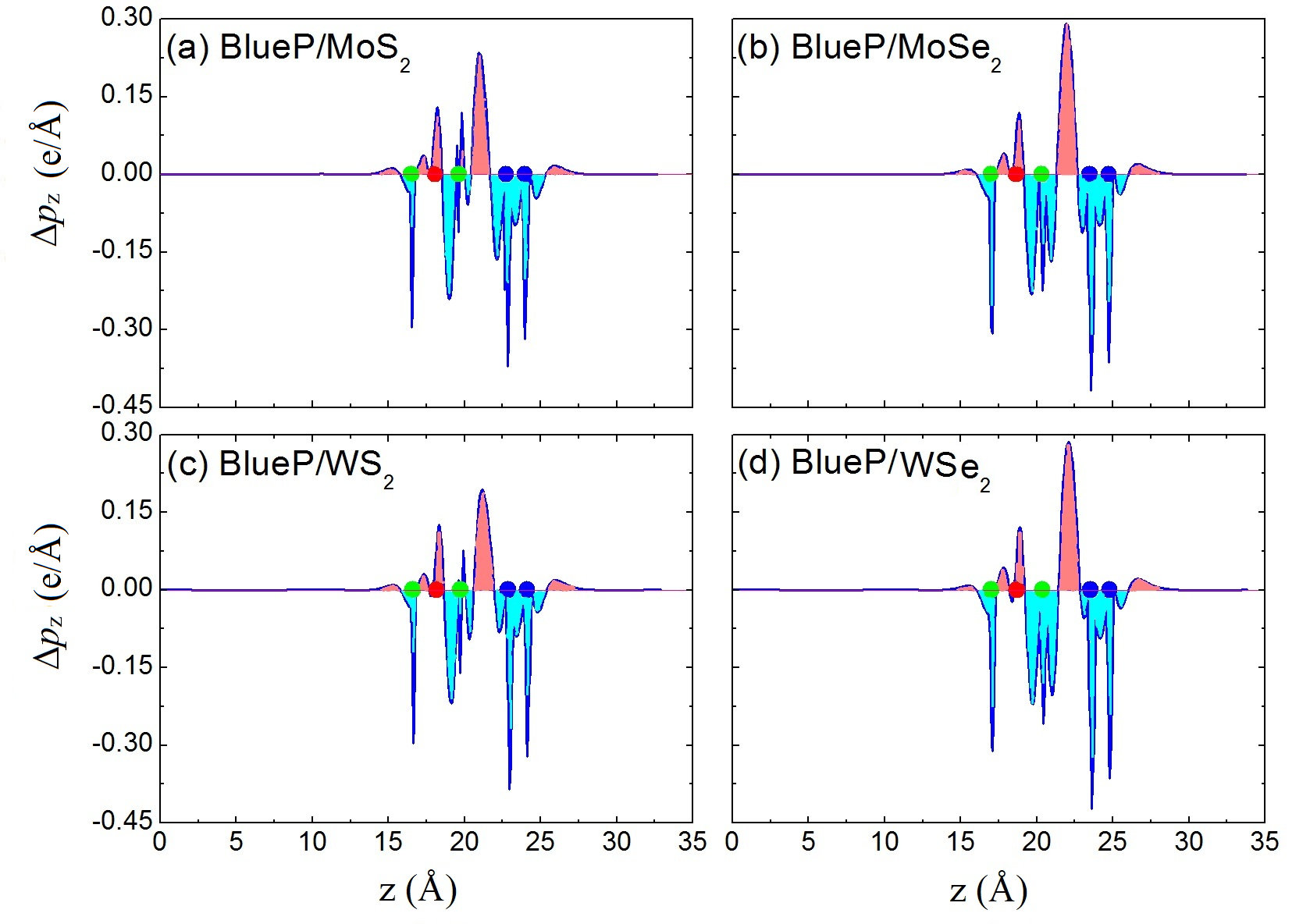


**Figure S7.** **Plane-averaged electron density difference along the direction perpendicular to the interface of (a) BlueP/MoS_2_, (b) BlueP/MoSe_2_, (c) BlueP/WS_2_ and (d) BlueP/WSe_2_ heterostructures, respectively.** The positions of the Mo (W), S (Se) and BlueP atoms are indicated by red, green and blue solid circles, respectively. The magenta and cyan colors indicate electron accumulation and depletion, respectively.


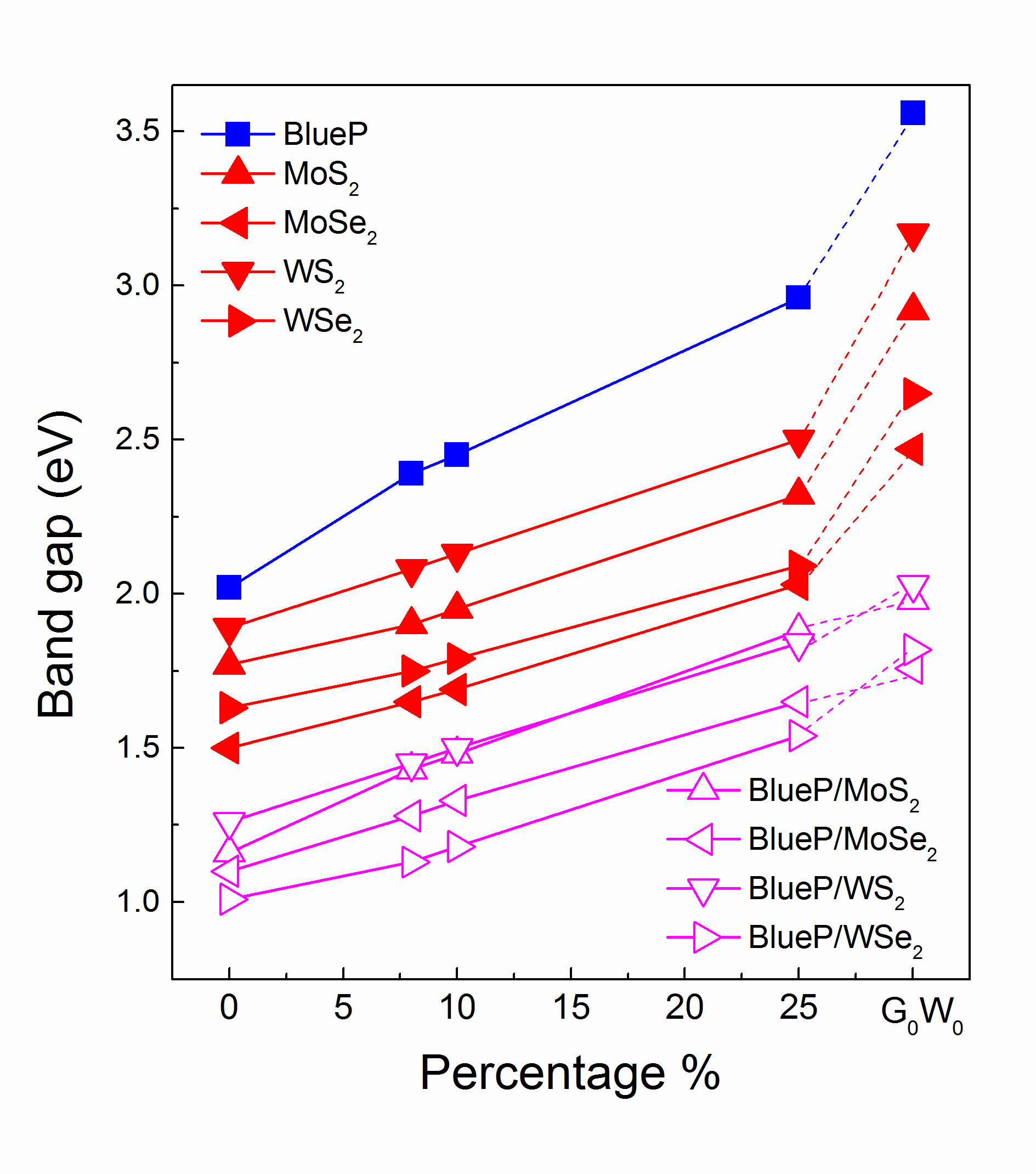


**Figure S8.** **The calculated band gap as a function of the short range Hartree-Fock exchange energy as well as the G_0_W_0_ approach based on optB86b-vdW for the monolayers and heterostructures.** The 0 % is the standard optB86b-vdW functional, and the 25 % presents the HSE06 hybrid functional.

**References**

1. Ghosh, B., Nahas, S., Bhowmick, S. & Agarwal, A. Electric field induced gap modification in ultrathin blue phosphorus. *Phys. Rev. B* 91, 115433, (2015).

2. Zhu, Z. & Tománek, D. Semiconducting layered blue phosphorus: a computational study. *Phys. Rev. Lett.* 112, 176802, (2014).

3. Huang, W., Luo, X., Gan, C. K., Quek, S. Y. & Liang, G. Theoretical study of thermoelectric properties of few-layer MoS_2_ and WSe_2_. *Phys. Chem. Chem. Phys.* 16, 10866-10874, (2014).

4. Liao, J., Sa, B., Zhou, J., Ahuja, R. & Sun, Z. Design of high-efficiency visible-light photocatalysts for water splitting: MoS_2_/AlN (GaN) heterostructures. *J. Phys. Chem. C* 118, 17594-17599, (2014).

5. Ding, Y. *et al.* First principles study of structural, vibrational and electronic properties of graphene-like MX_2_ (M= Mo, Nb, W, Ta; X= S, Se, Te) monolayers. *Physica B* 406, 2254-2260, (2011).

6. Shi, H., Pan, H., Zhang, Y. W. & Yakobson, B. I. Quasiparticle band structures and optical properties of strained monolayer MoS_2_ and WS_2_. *Phys. Rev. B* 87, 155304 (2013).

7. Mak, K. F., Lee, C., Hone, J., Shan, J. & Heinz, T. F. Atomically thin MoS_2_: a new direct-gap semiconductor. *Phys. Rev. Lett.* 105, 136805, (2010).

8. Korn, T., Heydrich, S., Hirmer, M., Schmutzler, J. & Schüller, C. Low-temperature photocarrier dynamics in monolayer MoS_2_. *Appl. Phys. Lett.* 99, 102109, (2011).

9. Zeng, H. *et al.* Optical signature of symmetry variations and dpin-valley coupling in atomically thin tungsten dichalcogenides. *Sci. Rep.* 3, 1608, (2013).

10. Zhang, Z. Y. *et al.* Bandgap engineering in van der Waals heterostructures of blue phosphorene and MoS_2_: A first principles calculation. *J. Solid State Chem.* 231, 64-69, (2015).
